# The Bridge Symptoms of Work–Family Conflict, Sleep Disorder, and Job Burnout: A Network Analysis

**DOI:** 10.1155/2024/2499188

**Published:** 2024-11-04

**Authors:** Jingyan Sun, Siyuan Wang, Ying Huang, Sze Tung Lam, Yixin Zhao, Yuqiong He, Hanrui Peng, Huijuan Guo, Xiaoping Wang

**Affiliations:** ^1^Department of Psychiatry, National Clinical Research Center for Mental Disorders, The Second Xiangya Hospital of Central South University, Changsha 410011, Hunan, China; ^2^Department of Psychiatry, Pingtang Compulsory Isolation Detoxification Institute in Hunan Province, Changsha, China; ^3^Saw Sweet Hock School of Public Health, National University of Singapore, 12 Science Drive 2, #10−01, Singapore 117549, Singapore

**Keywords:** bridge symptoms, correctional personnels, sleep disorder, symptom network, work–family conflict

## Abstract

**Background:** This study aims to elucidate characteristics of the symptom network of work–family conflict (WFC) and sleep disorders among Chinese correctional personnels while accounting for job burnout as a possible confounder.

**Method:** A total of 472 correctional personnel were included. Their WFC, sleep disorders, and job burnout were measured using a Chinese version of Work–Family Conflict Scale (WFCS), the Athens Insomnia Scale (AIS), and a revised Chinese version of Maslach Burnout Inventory-General Survey (MBI-GS), respectively. Central symptoms and bridge symptoms were respectively identified based on centrality indices and bridge centrality indices. Network stability was examined using the case-dropping procedure.

**Results:** Daytime condition (strength = 0.01) and strain-based work interference with family (WFCs, strength = 1.45) symptoms had the highest centrality values in the WFC-sleep disorder network structure, which were also identified as two bridge symptoms. Emotional exhaustion, daytime condition, and WFCs appeared to be potential bridge symptoms in the WFC–sleep disorder–burnout network structure.

**Conclusions:** In this study, among Chinese correctional personnel, daytime conditions, and WFCs were found to be central symptoms in the WFC–sleep disorder network structure, with emotional exhaustion as the bridge symptom in the WFC–sleep disorder–burnout network structure. We encourage relevant organizations to provide timely and effective education and guidance for regulatory personnel regarding these bridge symptoms. Subsequent research should follow up to assess the impact of relevant interventions on symptoms in the WFC–sleep disorder–burnout network, thereby advocating for the mental and physical health of correctional personnel.

## 1. Introduction

Compared to other professions, correctional personnel face more occupational risks and challenges and experience more work–family conflicts (WFCs) [1]. According to role conflict theory, the stress arising from the demands of work and family leads to WFC [2], which is defined as “a type of role conflict where the role pressures from work and family domains are incongruent.” [3]. Correctional personnel endure substantial workloads, inadequate time off work, and encounter challenges in reconciling work and family responsibilities, resulting in significant WFC [4]. Prior research has indicated that WFC is linked to sleep disorder, occupational burnout, and other mental health issues [5].

Sleep disorders are prevalent clinical conditions marked by challenges in initiating and maintaining sleep, along with associated symptoms, including irritability and fatigue during wakefulness [6]. Previous research has indicated a high prevalence of sleep disorders among correctional personnel, with some estimates reaching above 55%, which has a significant impact on mental health and productivity [7]. This is significantly associated with workplace factors, including work-related stress, aggressive and threatening behavior directed at correctional officers in the preceding 12 months, and physical activity related to work [7]. Several studies have identified a significant association between sleep disorders and WFC, with WFC impacting both the duration and quality of sleep [8]. A study of Italian nurses reveals that increased WFC is associated with a higher degree of sleep deprivation [9]. There is a suggestion of a dose–response relationship as well, with increasing levels of WFC leading to more pronounced sleep disorders [10]. While these studies have demonstrated, with strong evidence, the impact of WFC on healthcare professionals' sleep patterns, there is limited research pertaining to correctional personnel.

Burnout is a prolonged response to chronic emotional and interpersonal stressors at work, and it encompasses three dimensions, namely (1) emotional exhaustion, (2) depersonalization or cynicism, and (3) reduced personal accomplishment [11]. In China, there are approximately 300,000 prison guards for 1.64 million prisoners, resulting in a ratio of approximately 1:5 (“<Comparative studies of Chinese and foreign systems for prison officers.pdf>,”). Beyond the rigorous job requirements, career-related issues, such as effort-to-reward misalignment and uncertain promotion prospects, contribute to a relatively high prevalence of job burnout among regulatory personnel in China [12]. WFC is generally considered a long-term stressor. Research on correctional personnel indicates a positive correlation between WFC and fatigue [13] with a possible dose–response relationship. An observational study of newly hired American correctional officers suggests that increased WFC is associated with increased job burnout during their employment [14].

Previous research have also shown a close association between job burnout and sleep disorders [15]. Job burnout is considered a chronic syndrome resulting from stress in the workplace. A systematic review on job burnout revealed the deleterious effects of burnout on the physical and mental health on affected workers, with burnout being a strong predictor of insomnia and depressive symptoms [16]. The demanding nature of correctional work, including irregular work schedules, long shifts, and mandatory night shifts, could further exacerbate the risk of developing job burnout. [17].

Although several studies have explored the association between WFC, burnout, and sleep disorders, there is still a dearth of research in the specific group of correctional officers and a lack of more in-depth mechanistic studies. Based on the above research, we aim to investigate the impact of WFC, job burnout, and sleep disorders on correctional officers in China and then emphasize the important pathways and nodes therein. Owing to the heterogeneity of WFC and its effects, we chose conducted network analysis to explore interrelationships and evaluate the connectivity between the WFC structure and sleep disorders. In doing so, we aim to discover key WFC features with particular influence on the development of burnout, and recommend targeted interventions to prevent the development of burnout and its associated hazardous consequences.

Over the past decade, novel methods of network analysis have garnered considerable attention and have been widely applied to explore the core of psychological and therapeutic processes [18]. Moreover, Network analysis provides new insights into the functional roles and importance of symptoms in the persistence of diseases [19, 20]. It also allows for the identification of central and bridge symptoms within a network, enhancing the understanding of multidimensional relationships between commonly comorbid psychiatric symptoms and diagnoses [21].

In this study, we aim to, within the sample of Chinese correctional officers, (1) identify the central symptoms of WFC and sleep disorders, (2) investigate the bridge symptoms between WFCs and sleep disorders, and (3) evaluate the connectivity of WFCs and sleep disorders while considering job burnout as a possible confounder.

## 2. Methods

### 2.1. Subjects

The cross-sectional study was carried out from October 2021 to January 2022, involving correctional officers from various provinces in China. An anonymous online survey was developed using Questionnaire Star (https://www.wjx.cn), a widely utilized online survey platform in China. Initially, 10 correctional officers who participated in a training course were chosen as “original distributors.” Subsequently, we employed a snowball sampling method, where the link to the online survey was disseminated through popular social media platforms such as WeChat by the original distributors to their friends and WeChat group members.

On the initial page of the questionnaire, a concise overview of the study and assurances of anonymity and confidentiality were given. All participants acknowledged and provided informed consent by clicking the “agree” button before proceeding to answer the questions. They were also given the option to click “disagree” in order to exit the survey or to withdraw at any point. This study was approved by the Ethics Committee of the Second Xiangya Hospital of Central South University.

### 2.2. Sociodemographic Data

Sociodemographic data was collected using a self-designed questionnaire, which included age, gender, education, years worked as a correctional officer, marital status, and location of practice.

### 2.3. Clinical Data

Clinical data, including sleep disorder, WFC, and job burnout, were assessed using the aforementioned scales.

#### 2.3.1. Athens Insomnia Scale (AIS)

The AIS was used to assess the insomnia status of the subjects [22]. The scale comprises eight items, with the initial five assessing sleep quality and the remaining three evaluating the daytime condition of the subjects with insomnia. A 4-point scale was employed to gauge the severity of the subjects' insomnia. It has a total score range of 0–24, where a score lower than four indicates no insomnia, a score of 4–6 indicates suspected insomnia, and a score exceeding six suggests the presence of insomnia. In this study, the Cronbach's *α* coefficient for this scale was 0.900.

#### 2.3.2. Work–Family Conflict Scale (WFCS)

The Chinese version of WFCS was used to assess the WFC experienced by the participants [23]. The WFCS comprises 18 items, with two subsections: work interference with family and family interference with work. All items were assessed using a 5-point Likert scale (ranging from 1 = never to 5 = always), with 18 being the minimum score, 90 being the maximum, and the score being proportional to the WFC experienced. In this study, the Cronbach's *α* coefficient for this scale was 0.917.

#### 2.3.3. Maslach Burnout Inventory-General Survey (MBI-GS)

A revised Chinese version of MBI-GS was used to assess the participants' burnout [24]. This assessment consists of 15 items assessing three dimensions of burnout: emotional exhaustion, depersonalization and reduced personal accomplishment. Each item was evaluated on a 7-point Likert scale (ranging from 0 = never to 6 = every day), with five items in the personal accomplishment section being scored in reverse. The total score is computed by summing all item scores, then dividing by 15 and multiplying by 20, resulting in a modified score range of 0–120. A higher score on this scale denotes more significant burnout. In this study, the Cronbach's *α* coefficient for this scale was 0.830.

### 2.4. Theoretical Framework

The stress–strain model assumes that stressors (external pressures or demands) lead to stress (internal reactions or symptoms), which in turn lead to a variety of negative outcomes, and can be used to explain the association between WFC, sleep disorder, and job burnout. WFC is a significant stressor that arises when the demands of work and family roles are incompatible, making it difficult for individuals to fulfill their responsibilities. Conflict is a significant stressor that arises when the demands of work and family roles are incompatible, making it difficult for individuals to fulfill both roles effectively [25]. The stress of juggling work and family responsibilities can disrupt sleep patterns, leading to consequences such as poor sleep quality and insomnia, and the resulting decline in daytime functioning can be a new source of stress [26]. Job burnout is a common consequence of long-term exposure to WFC [25], while sleep disorders can exacerbate feelings of exhaustion and decrease an individual's ability to cope with daily stressors [27].

### 2.5. Statistical Analyses

To ensure the quality of our surveys, all completed questionnaires were screened for inappropriate responses and lack of variation in responses to open-ended questions. Those responses that did not correctly answer validity entries, had a scale completion time of <1 min, or had too much option homogeneity, were excluded. We used SPSS 26.0 software to process missing values (multiple interpolations), statistical sociodemographic information, and scale scores. Gender differences in scale scores were verified by nonparametric tests.

### 2.6. Network Estimation

Network models were performed on the R software (version 4.2.2) using the qgraph package (version 1.9.2) [28, 29]. The method of partial correlation network was employed to estimate all symptomatic networks, using the least absolute shrinkage and selection operator (LASSO) technique for regularization [30]. This approach simplifies the results and yields interpretable network models. The tuning parameter for LASSO was selected based on the extended Bayesian information criterion (EBIC) and finalized within the framework of the Gaussian graphical model [31]. Given the ordinal nature of the data, Spearman's correlation was applied.

In this network, each node represents a symptom from the AIS, WFCS, or MBI-GS scales. Edges between nodes depict dependencies, with blue indicating positive associations and red indicating negative ones. Thicker edges signify stronger associations. Abbreviations were assigned to the two AIS, six WFCS, and three MBI-GS dimensions and are reflected in figures showing node centrality values.

### 2.7. Network Central and Bridge Symptom Estimation

Three standard centrality metrics were applied in the study—strength, closeness, and betweenness—to assess node characteristics [32]. “Strength” refers to the total weight of connections a node receives, representing its overall symptom importance. “Closeness” is the inverse of the sum of the shortest path lengths from a node to all others, indicating its proximity to other symptoms. “Betweenness” captures how frequently a node lies on the shortest path between two other symptoms, highlighting its role in mediating associations. Bridge symptoms, seen as cross-diagnostic markers, may serve as effective intervention points for multiple psychiatric disorders [32].

### 2.8. Network Accuracy and Stability Estimation

We employed the case-dropping method to assess edge accuracy and network stability, utilizing the bootnet R package (version 1.5) over 1000 iterations [31]. Additionally, we referenced the centrality stability coefficient (CS-coefficient) as a benchmark. A CS-coefficient below 0.25 indicated considerable instability, while values of 0.5 or higher were deemed ideal.

## 3. Results

A total of 472 correctional officers were included, and the demographic characteristics of all participants are shown in Table 1. Of these, males and married correctional officers were in the majority. About 56.1% of correctional officers experienced insomnia. Correlation analysis showed (Table 2) that there was a very strong correlation between WFC, sleep disorders, and burnout, which was consistent with the hypotheses proposed in the previous section and laid the foundation for further network analysis.

### 3.1. Model 1: Psychopathology of Insomnia and WFC

**Figure 1:**
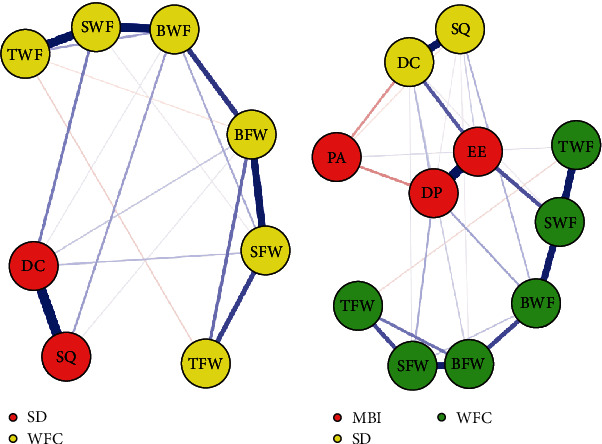
Results of network analysis. The insomnia and WFC network (A), and the insomnia, WFC, and burnout network (B). *Note:* Labels for insomnia: DC, daytime condition; SQ, sleep quality. Labels for WFC: BFW, behavior-based family interference with work; BWF, behavior-based work interference with family; SFW, strain-based family interference with work; SWF, strain-based work interference with family; TFW, time-based family interference with work; TWF, time-based work interference with family. Labels for burnout: DP, depersonalization; EE, emotional exhaustion; PA, personal accomplishment. Strength is indicated by the thickness of lines between nodes, with thicker lines representing stronger ties. WFC, work–family conflict.

**Figure 2:**
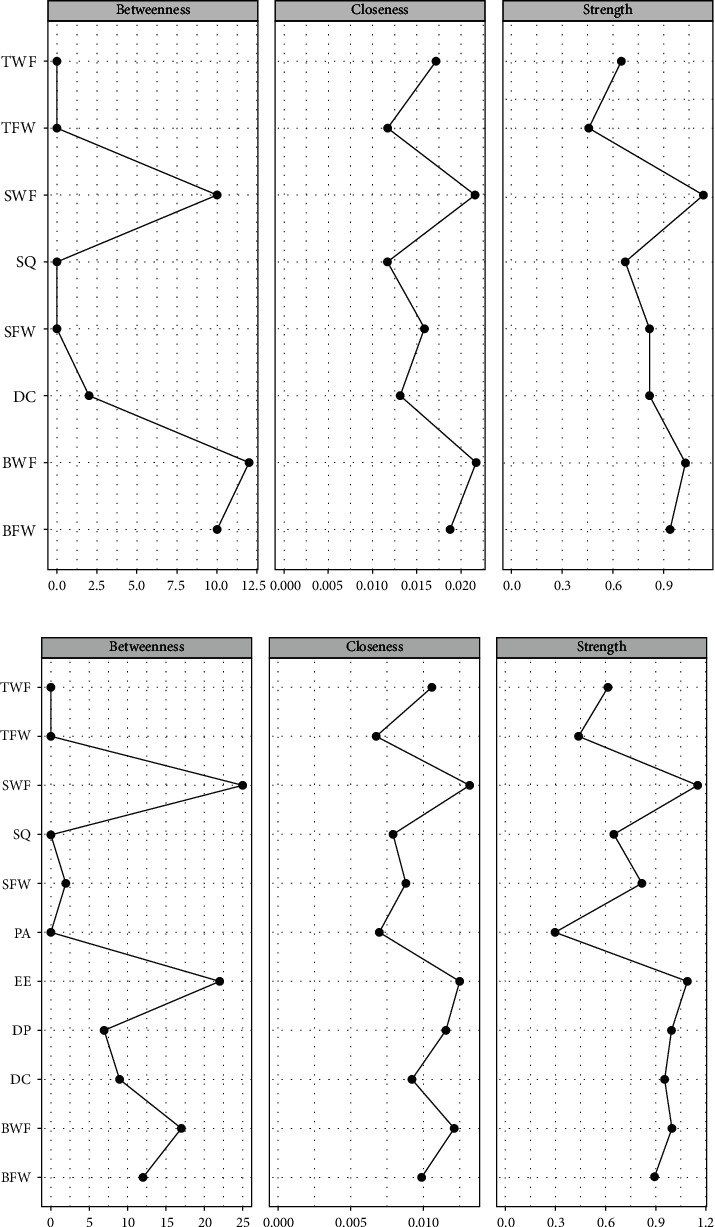
Centrality indices of the insomnia and WFC network, the insomnia, WFC, and burnout network. *Note:* Values shown on the *x*-axis are standardized *z*-scores. WFC, work–family conflict.

**Figure 3:**
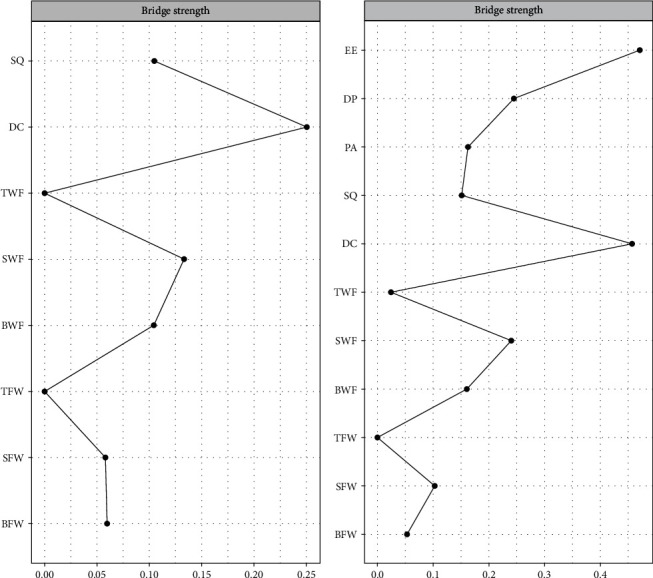
Bridge centrality indices of the insomnia and WFC network, the insomnia, WFC, and burnout network. *Note:* Labels for insomnia: DC, daytime condition; SQ, sleep quality. Labels for WFC: BFW, behavior-based family interference with work; BWF, behavior-based work interference with family; SFW, strain-based family interference with work; SWF, strain-based work interference with family; TFW, time-based family interference with work; TWF, time-based work interference with family. Labels for burnout: DP, depersonalization; EE, emotional exhaustion; PA, personal accomplishment. Strength is indicated by the thickness of lines between nodes, with thicker lines representing stronger ties. WFC, work–family conflict.

Two clusters of symptoms (insomnia and WFC symptoms) were found to be bridged by several symptoms (Figure 1A). The insomnia symptoms that are closest to WFC symptoms were as follows (with WFC symptoms in parentheses): daytime condition (close to strain-based work interference with family [WFCs]) and sleep quality (close to behavior-based work interference with family) (see Table S1 and Figure 2A). The insomnia symptom with the highest centrality was daytime condition (strength = 0.01), whereas the WFC symptoms with the behavior-based work interference with family (betweenness = 1.43, closeness = 1.29, strength = 0.97) and WFCs betweenness = 1.07, closeness = 1.26, strength = 1.45). According to their respective bridge strengths, daytime condition and WFCs were the two most prominent bridge symptoms in this model (Figure 3A).

### 3.2. Model 2: Psychopathology of Insomnia, WFC, and Burnout

Three clusters of symptoms (insomnia, WFC, and burnout symptoms) were found to be bridged by several symptoms (Figure 1B). The burnout symptoms closest to insomnia symptoms and WFC symptoms are as follows (with insomnia symptoms and WFC symptoms in parentheses): emotional exhaustion (close to daytime condition and WFCs) and depersonalization (close to daytime condition and behavior-based work interference with family) (see Table S2 and Figure 2B). The insomnia symptom with the highest centrality was daytime condition (strength = 0.53), the WFC symptoms with the highest centrality were WFCs (betweenness = 1.76, closeness = 1.46, and strength = 1.24) and behavior-based work interference with family (betweenness = 0.91, closeness = 0.97, and strength = 0.69), whereas the burnout symptoms with the highest centrality were emotional exhaustion (betweenness = 1.44, closeness = 1.14, and strength = 1.02) and depersonalization (closeness = 0.71, and strength = 0.68). According to the bridge strength, emotional exhaustion, daytime condition, and WFCs were the three most prominent bridge symptoms in this model (Figure 3B).

### 3.3. The Impact of Gender

The literature suggests that women are often expected to juggle work and family at the same time and, therefore, often experience more WFC [33] and that the prevalence of insomnia is higher among women [34]. To exclude the effect of gender, we used nonparametric tests to compare the different genders in terms of scale score differences (Table 3), and network analyses were performed separately for each gender (Figures S1 and S2). The results did not show gender differences.

### 3.4. Stability Analyses

**Figure 4:**
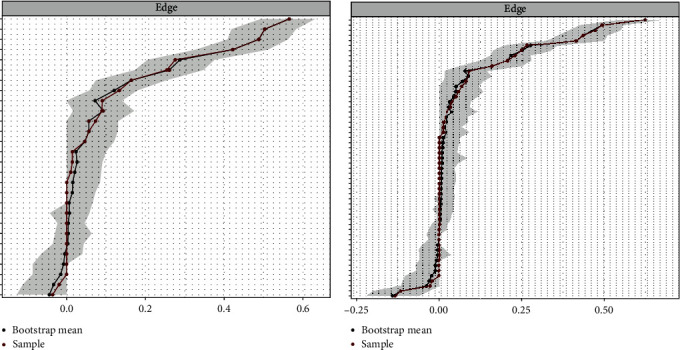
Accuracy of edge weight in the insomnia and WFC network, the insomnia, WFC, and burnout network. WFC, work–family conflict.

The stability analyses showed that the network models were stable (Figure 4). Specifically, the edge weight stability analyses suggested that the tie strengths were reliably estimated. The node-dropping stability analyses suggested that the order of the nodes with regard to centrality was stable even after removing up to 50% of the nodes in each network. Strength centrality appeared to be the most stable among the centrality measures.

## 4. Discussion

To our knowledge, this is the first study to describe the network of symptoms of WFC and sleep disorder. We identified several core symptoms and bridge symptoms between WFC and the group of sleep disorder symptoms, as well as bridge symptoms between WFC, sleep disorders, and the group of job burnout symptoms. Overall, from the results of these network analyses, we arrived at two common findings. First, in all networks, the core symptoms of sleep disorders and WFC are the daytime conditions and WFCs, and in terms of the overlap between sleep disorders and WFC, it was found that the daytime state and WFCs are bridge symptoms connecting WFC and sleep disorders. Second, in terms of the overlap between WFC and sleep disorders and job burnout, emotional exhaustion, daytime condition, and WFCs were the most prominent bridge symptoms in this model.

Within the framework of the network model of WFC and sleep disorder, it has been established that the central symptoms include daytime conditions and WFCs. Research among Malaysian civil servicewomen demonstrated that higher levels of WFCs were linked to a rise in the occurrence and severity of sleep disturbances [35]. A different study has shown that WFCs correlate with sleep problems in cross-sectional and longitudinal follow-up data [36]. A longitudinal study surveying the use of sedative-hypnotic drugs and WFC in women indicates that the conflict between family and work is associated with the use of sedative drugs in the past 5 years. The link between WFCs and sleeping pills is stronger than the association between time-based WFC and sleeping pills [37]. Poor sleep quality can lead to decreased condition and increase the risk of physical and mental illness [38, 39]. The prevalence of sleep disorders plays a key role in diminishing the quality of life. Research on Parkinson's patients revealed a high prevalence of comorbid sleep disorders, significantly impacting their quality of life [40]. Insomnia among correctional personnel can contribute to impaired daytime function and various health hazards, including job burnout, daytime sleepiness, irritability, and low mood, leading to decreased work efficiency [16, 41]. Our findings indirectly suggest that WFCs and daytime conditions are pivotal in the network of WFC and sleep disorders. These two key symptoms are anchored at the core of the network and exhibit the most robust and frequent connections with other symptoms of WFC and sleep disorders.

Bridge symptoms are regarded as cross-diagnostic indicators in clinical settings, thus, interventions directed towards these symptoms could prove effective in addressing multiple psychiatric conditions concurrently [42]. However, the current research methodology is predominantly cross-sectional, and the results of these studies merely suggest a link between WFC and sleep problems. Due to a lack of follow-up over time, the potential mechanisms by which WFC leads to sleep disturbances remain unclear. The network analysis conducted in this study revealed that the most prominent connecting symptoms of WFC and sleep disorder are impaired daytime conditions and WFCs. This could be attributed to WFCs, which is a form of physiological stress from the external environment, potentially interfering with and dysregulating the hypothalamic–pituitary–adrenal (HPA) axis [43]. WFCs, as a form of chronic stress, can affect the overall diurnal pattern of cortisol secretion, leading to higher levels of cortisol at night, which can interfere with sleep and contribute to sleep disorders [43, 44]. Furthermore, Melatonin is a hormone produced by the pineal gland that regulates sleep–wake cycles. Stress from WFC can suppress melatonin production, leading to difficulties in falling asleep and maintaining sleep, thereby contributing to insomnia [45]. The combination of WFC and sleep disorders creates a vicious cycle. Stress from WFC disrupts sleep, and poor sleep quality further diminishes the ability to manage work and family demands, leading to increased stress and eventual burnout [46].

In previous studies, not only have WFC and sleep disturbances been shown to be associated with job burnout [14, 47], but these factors may even mutually influence each other [48]. A study among Korean nurses found that they experience significant burnout, with sleep disturbances mediating the relationship between WFC and burnout [5], supporting our findings. Another study examining the dimensions of burnout and WFC among Chinese physicians and their coping strategies revealed that WFC and family interference with work were associated with different dimensions of job burnout, whereas coping strategies acted as a mediator of burnout [49]. Similarly, a study of Canadian nurses revealed that emotional exhaustion, a key aspect of burnout, was partially predictive of WFC and was a key link between WFC and job demands. Burnout can markedly impair employees' work quality. A study on Chinese interns indicated a significant negative correlation between the occurrence of burnout and organizational fairness, impacting healthcare quality [50].

To validate this perspective, we developed a psychopathological network model encompassing WFC, sleep disorder, and job burnout and identified emotional exhaustion, condition, and WFCs as potential bridge symptoms linking WFC, sleep disorder, and job burnout. With heightened work pressure, correctional personnel are predisposed to emotional exhaustion. Prior studies have demonstrated a close association between WFC and emotional exhaustion. As WFC increases, the level of emotional exhaustion similarly rises [9].

During the pandemic, the workload of correctional personnel intensified, thereby increasing occupational demands and workplace stress, which in turn results in emotional exhaustion and further detriment to mental health [51, 52]. This has led to a decline in nighttime sleep quality and daytime conditions. Moreover, the decrease in daytime conditions has reduced work enthusiasm, thereby worsening emotional exhaustion. A study on frontline healthcare workers in Poland during the COVID-19 pandemic revealed that coronavirus-related anxiety contributed to sleep disorder, ultimately leading to emotional exhaustion [53]. Prior studies have demonstrated a positive correlation between the severity of sleep disorders and emotional exhaustion [54]. In conclusion, our study suggests that emotional exhaustion may play a moderating role in regulating the relationship between WFC and sleep disorders.

This study represents the first research on the network analysis of WFC-sleep disorders within the Chinese correctional population, yet it has some limitations. First, our study used a snowball sampling method, included a small population, and the utilization of a cross-sectional design prevents the revelation of the causal relationship and corresponding dynamic changes between WFC, sleep disorders, and job burnout. Multicenter cohort studies should be considered in the future, and attention should be paid to the use of random sampling. Another limitation of this study is the focus on Chinese correctional personnel; these results could only be generalized to the occupational health of Chinese correctional personnel. Caution should be made when extrapolating these findings to other populations, given potential differences in contextual and occupational settings. In addition, the healthy worker effect may also have an influence on our research [55]; we may have missed those whose condition is so much more severe that they cannot work. In the future, a wider range of follow-up studies should be considered. Lastly, the article only reports the results of an observational study; interventions targeting bridge symptoms should be considered in the future and observed for efficacy.

## 5. Conclusion

In conclusion, we found that daytime conditions and WFCs were core symptoms of sleep disorders and WFC network models, while daytime conditions and WFCs serve as bridge symptoms. We also found that emotional exhaustion, daytime condition, and WFCs were potential bridge symptoms connecting WFC, sleep disorders, and job burnout. These findings have pertinent implications on subsequent occupational health interventions; relevant occupational health and professional organizations should provide effective knowledge dissemination and intervention guidance for correctional officers. For the improvement of correctional personnels' mental health, we suggest that to maximize the impact of proposed interventions, they should aim to improve nighttime sleep quality, balance work–family relationships, and facilitate timely detection and reduction of emotional exhaustion. Subsequent research should follow up to verify whether interventions targeting the related bridge symptoms can improve other symptoms in the WFC-sleep disorder network.

## Figures and Tables

**Table 1:** Demographic characteristics (*n* = 472).

Variables	M (SD) or *N* (%)
Age (years)	37.63 (8.10)
Gender	
Female	150 (31.8)
Male	322 (68.2)
Education (years)	16.04 (0.69)
Years worked as a correctional officer	13.58 (9.58)
Marriage	
Married	379 (80.3)
Unmarried	71 (15.0)
Divorced	22 (4.7)
Location of practice	
Platoon and the front line	324 (68.6)
Department and secretary staff	148 (31.4)

Abbreviations: M, mean; SD, standard deviation.

**Table 2:** Descriptive statistics and bivariate correlations.

Facotors	M± SD	WFCS	AIS	MBI-GS
WFCS	60.45 ± 12.90	1	—	—
AIS	7.81 ± 4.77	**0.40** *⁣* ^*∗∗∗*^	1	—
MBI-GS	51.47 ± 27.00	**0.50** *⁣* ^*∗∗∗*^	**0.54** *⁣* ^*∗∗∗*^	1

*Note:* Results in bold are statistically significant.

Abbreviations: AIS, Athens Insomnia Scale; M, mean; MBI-GS, Maslach Burnout Inventory-General Survey; SD, standard deviation; WFCS, Work–Family Conflict Scale.

*⁣* ^*∗∗∗*^ *P* < 0.001.

**Table 3:** Gender differences in scale scores.

Scale	Male	Female	*Z*	*P*
WFCS	61.18 ± 13.36	58.90 ± 11.75	−1.846	0.06
AIS	7.98 ± 4.86	7.45 ± 4.57	−1.417	0.16
MBI-GS	52.52 ± 27.55	49.19 ± 25.72	−1.207	0.23

*Note:* Scale scores are expressed as mean ± standard deviation.

Abbreviations: AIS, Athens Insomnia Scale; MBI-GS, Maslach Burnout Inventory-General Survey; WFCS, Work–Family Conflict Scale.

## Data Availability

The raw data supporting the conclusions of this article will be made available by the authors without undue reservation.

## Nomenclature

WFC: Work–family conflict
WFCS: Work–Family Conflict Scale
AIS: The Athens Insomnia Scale
MBI-GS: Maslach Burnout Inventory-General Survey (MBI-GS)
WFCs: Strain-based work interference with family
EBIC: The extended Bayesian information criterion
HPA: Hypothalamic–pituitary–adrenal.

## Supporting Information

Supporting Information The strength of edges in insomnia and work–family conflict network are shown in Table S1: the partial correlation coefficient between daytime condition (DC) and strain-based work interference with family (SWF) is 0.13, and the partial correlation coefficient between sleep quality (SQ) and Behavior based family interference with work (BWF) is 0.13 (Table S1). The strength of connections in the network involving insomnia, work–family conflict, and burnout is presented in Table S2: the partial correlation coefficients are detailed as follows: emotional exhaustion (EE) and depersonalization (DC) (0.21), EE and strain-based work interference with family (SWF) (0.23), depersonalization (DP) and DC (0.06), DP and behavior-based work interference with family (BWF) (0.09) (Table S2). The network analysis results for males are shown in Figure S1: and for females are shown in Figure S2: the strongest edge weights are found in both genders between emotional exhaustion and depersonalization, strain-based family interference with work, and behavior-based family interference with work, strain-based work interference with family and time-based work interference with family, daytime sleep conditions and sleep quality. Nonetheless, the results revealed no gender discrepancies (Figures S1 and S2).
