# Supplementary material for: The Bridge Symptoms of Work–Family Conflict, Sleep Disorder, and Job Burnout: A Network Analysis
Source: Depress Anxiety. 2024 Nov 4;2024:2499188. doi: 10.1155/2024/2499188 (PMC11918927; doi:10.1155/2024/2499188)
Supplement: Supporting Information — The strength of edges in insomnia and work–family conflict network are shown in Table S1: the partial correlation coefficient between daytime condition (DC) and strain-based work interference with family (SWF) is 0.13, and the partial correlation coefficient between sleep quality (SQ) and Behavior based family interference with work (BWF) is 0.13 (Table S1). The strength of connections in the network involving insomnia, work–family conflict, and burnout is presented in Table S2: the partial correlation coefficients are detailed as follows: emotional exhaustion (EE) and depersonalization (DC) (0.21), EE and strain-based work interference with family (SWF) (0.23), depersonalization (DP) and DC (0.06), DP and behavior-based work interference with family (BWF) (0.09) (Table S2). The network analysis results for males are shown in Figure S1: and for females are shown in Figure S2: the strongest edge weights are found in both genders between emotional exhaustion and depersonalization, strain-based family interference with work, and behavior-based family interference with work, strain-based work interference with family and time-based work interference with family, daytime sleep conditions and sleep quality. Nonetheless, the results revealed no gender discrepancies (Figures S1 and S2). [file 2499188.f1.docx]

Table S1. Strength of edges in the insomnia and work-family conflict network

| Edge | Partial correlation coefficient |
| --- | --- |
| DC-SWF | 0.13 |
| SQ-BWF | 0.09 |
| DC-SFW | 0.06 |
| DC-BFW | 0.05 |

Note: Edges were included only if the absolute value of the edge was equal to or greater than the cutoff score calculated for the glasso network (0.03). The cutoff score represents a strength greater than that of 75% of the edges. Labels for insomnia: SQ=Sleep quality; DC=Daytime condition; Labels for work-family conflict: TWF= Time based work interference with family; SWF =Strain based work interference with family; BWF=Behavior based work interference with family; TFW=Time based family interference with work; SFW=Strain based family interference with work; BFW=Behavior based family interference with work.

Table S2. Strength of edges in the insomnia, work-family conflict network and burnout

| Edge | Partial correlation coefficient |
| --- | --- |
| EE-SWF | 0.23 |
| EE-DC | 0.21 |
| DP-BWF | 0.09 |
| DP-SFW | 0.08 |
| DP-DC | 0.06 |
| EE-SQ | 0.03 |

Note: Edges were included only if the absolute value of the edge was equal to or greater than the cutoff score calculated for the glasso network (0.03). The cutoff score represents a strength greater than that of 75% of the edges. Labels for insomnia: SQ=Sleep quality; DC=Daytime condition; Labels for work-family conflict: TWF= Time based work interference with family; SWF =Strain based work interference with family; BWF=Behavior based work interference with family; TFW=Time based family interference with work; SFW=Strain based family interference with work; BFW=Behavior based family interference with work. Labels for burnout: EE=emotional exhaustion; DP=depersonalization; PA=personal accomplishment.


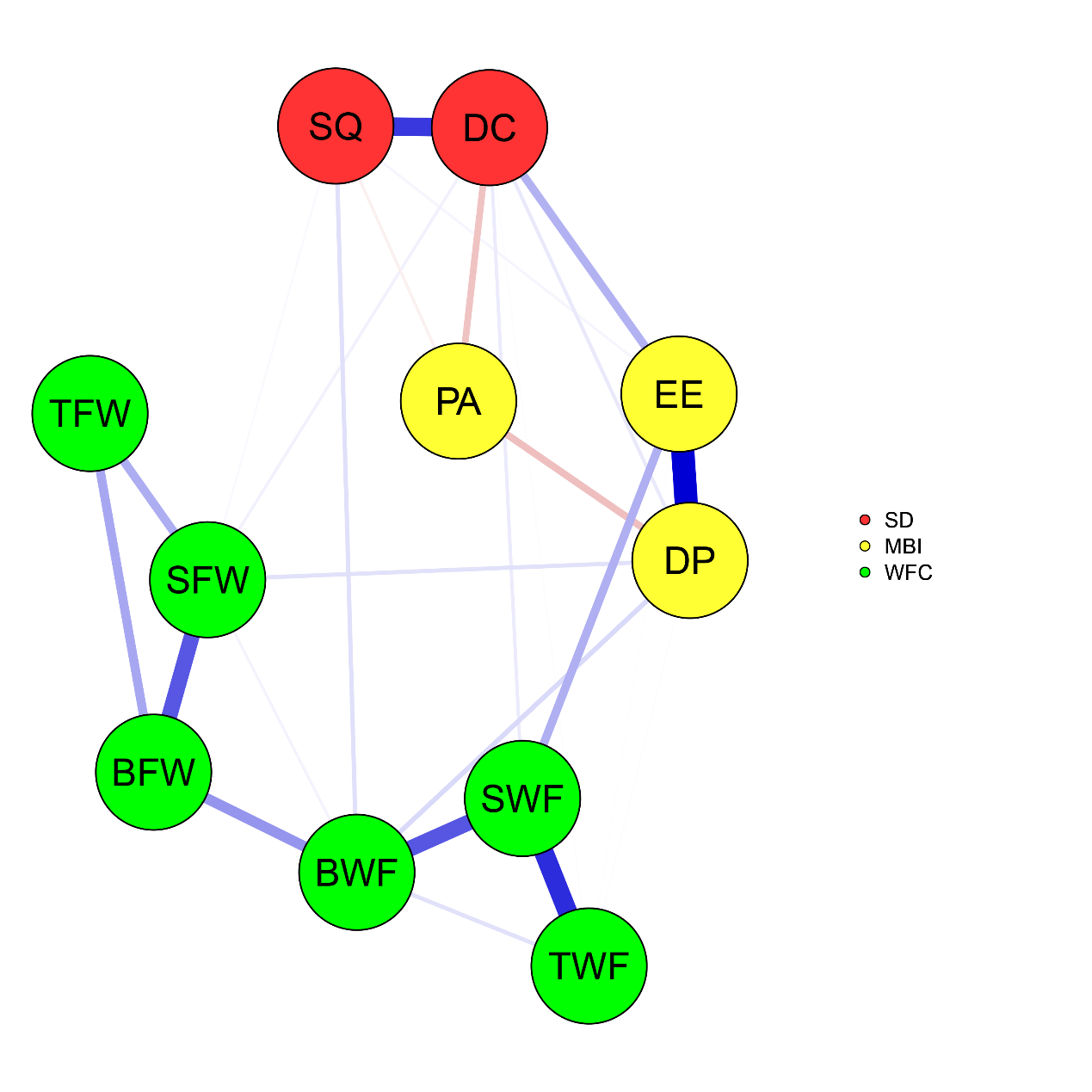


Figure S1. The insomnia, work-family conflict and burnout network for males.

Note. Labels for insomnia: SQ=Sleep quality; DC=Daytime condition; Labels for work-family conflict: TWF= Time based work interference with family; SWF =Strain based work interference with family; BWF=Behavior based work interference with family; TFW=Time based family interference with work; SFW=Strain based family interference with work; BFW=Behavior based family interference with work. Labels for burnout: EE=emotional exhaustion; DP=depersonalization; PA=personal accomplishment. Strength is indicated by the thickness of lines between nodes, with thicker lines representing stronger ties.


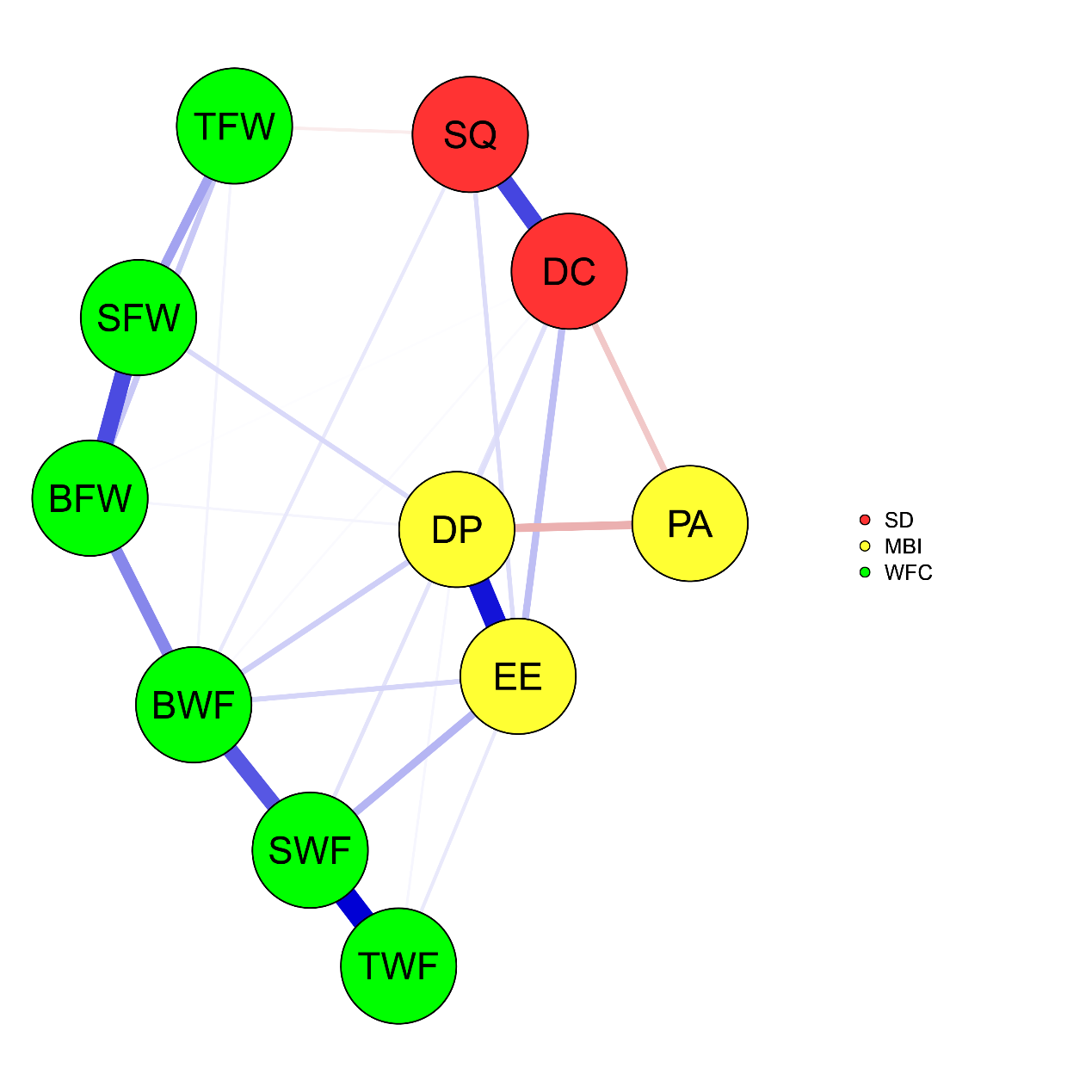


Figure S2. The insomnia, work-family conflict and burnout network for females.

Note. Labels for insomnia: SQ=Sleep quality; DC=Daytime condition; Labels for work-family conflict: TWF= Time based work interference with family; SWF =Strain based work interference with family; BWF=Behavior based work interference with family; TFW=Time based family interference with work; SFW=Strain based family interference with work; BFW=Behavior based family interference with work. Labels for burnout: EE=emotional exhaustion; DP=depersonalization; PA=personal accomplishment. Strength is indicated by the thickness of lines between nodes, with thicker lines representing stronger ties.
